# Dietary fiber in poultry nutrition: impacts on physiology, digestive metabolism and on productive performance

**DOI:** 10.1007/s11250-026-04970-6

**Published:** 2026-03-05

**Authors:** Marcelo Suzuki Suyama, Clóvis Eliseu Gewehr

**Affiliations:** Department of Animal Production, Center of Sciences, Agroveterinarias of University of Santa Catarina State (UDESC), Lages, Santa Catarina 88520-00 Brazil

**Keywords:** Digestibility, Enzyme, Gastrointestinal tract, Non-starch polysaccharides

## Abstract

Dietary fiber (DF) consists of non-starch polysaccharides (NSPs) and lignin, and is categorized into soluble and insoluble DF. DF is often regarded as an antinutritional element; however, the intake of both soluble and insoluble fiber can induce physiological alterations in the gastrointestinal tract (GIT). These adaptations include increased synthesis and secretion of digestive enzymes, altered gastrointestinal motility and development, improved gizzard functionality, and the proliferation of intestinal and caecal microbiota. The induced adaptations interfere with the digestibility of energy and nutrients in diets, which can affect the productive performance of the birds. Insoluble DF exhibit more beneficial effects on the GIT than soluble fibers, potentially enhancing the secretion of hydrochloric acid, pepsinogen, and cholecystokinin. The soluble fraction causes detrimental effects by increasing digesta viscosity and bacterial fermentation in the small intestine, which are exacerbated with increased soluble PNAs. This can be mitigated using exogenous carbohydrase enzymes that hydrolyze the β-bonds of the polysaccharide chain. This review aims to clarify the effect of DF on poultry diets, describe the effects caused in the GIT and digestion physiology by the soluble and insoluble fractions, how the use of carbohydrases can be a very efficient tool in reducing the antinutritional effects of NSPs, and the impacts of using fiber-rich feedstuffs on the productive performance of poultry.

## Introduction

Dietary fiber (DF) is considered an antinutritional component and a diluent in poultry diets, as evidenced by the negative correlation between elevated fiber content and the digestibility of energy, protein, starch, and ether extract (EE) in the diet (Smits and Annison 1996; Jha and Mishra 2021). An increase in fiber consumption can decrease the digestibility and absorption of nutrients owing to the difficulties associated with degrading non-starch polysaccharides (NSPs).

The incorporation of fiber-rich feedstuffs into the diet promotes various alterations in the gastrointestinal tract (GIT) of poultry, attributable to the effects of soluble and insoluble NSPs. The digestive organs experience adaptations in both weight and length to improve the digestive efficiency of DF, with these changes in the GIT differing based on the type of NSP. Furthermore, fiber exerts a prebiotic effect by promoting the proliferation of cecal bacterial colonies, enhancing the synthesis of short-chain fatty acids, lowering intestinal pH, and competitively inhibiting pathogenic bacteria.

Excessive soluble NSPs adversely affect nutrient digestibility by increasing viscosity and bacterial fermentation in the small intestine, particularly in the ileum. Nonetheless, the application of exogenous enzymes, such as carbohydrases, can mitigate these adverse effects (Choct 2006). Conversely, insoluble fibers can enhance nutrient utilization through gizzard adaptation (Abdollahi et al. 2019), increased digestive secretions (Svihus et al. 2004; Yokhana et al. 2016), and caecum adaptation in size and fermentative activity by the caecal microbiota (Röhe et al. 2020). The alterations induced by fiber in the poultry GIT’s directly influence the digestibility of dietary nutrients and, subsequently, the productive performance.

The impacts of DF consumption manifest throughout all phases of poultry life. Adult birds possess a superior ability to obtain energy from fiber-rich feedstuffs compared to young birds, attributable to their fully developed GIT and adaptations that enhance the digestion of such feedstuffs (Mateos et al. 2019). Despite the advantages of dietary fiber, its inclusion in poultry diet formulations is not prioritized due to its correlation with reduced digestibility. Consequently, minimum inclusion of fibrous ingredients is recommended to reduce their antinutritional effects. However, moderate additions of fiber, derived from both soluble and insoluble sources, can improve dietary digestibility by promoting adaptation of GIT.

This review aims to provide a comprehensive analysis of the effects of DF on poultry nutrition, including its influence on GIT adaptations, nutrient digestibility, the use of exogenous carbohydrases in NSP-rich diets, and the implications for poultry productive performance.

## Characterization of dietary fiber

The fibrous component of feedstuff is described as DF, consisting of several different NSPs and lignin. DF predominantly occurs in the plant cell wall, which consists of NSPs, lignin, and additional constituents, including proteins and fatty acids (McDougall et al. 1996). The primary polysaccharides constituting the cell wall are cellulose, hemicellulose, and lignin (Theander et al. 1989). Hemicellulose comprises a range of polysaccharides, including xylans, arabinoxylans, mixed-linkage β-glucans, xyloglucans, mannans, galactomannans, galactans, and arabinans (Choct 2015).

The NSPs consist of cellulose, hemicellulose, pectins, and fructans (Raza et al. 2019; Jha and Mishra 2021). NSPs contain beta bonds in their molecular structure and are not completely digestible, however, they are fermented by gut microbiota. DF can be categorized into soluble and insoluble based on their water solubility (Choct et al. 1996).

Barley and oats exhibit elevated levels of β-glucans, while wheat and rye are rich in arabinogalactans (Jha and Mishra 2021), and pectins are predominantly found in fruits and sugar beet pulp (González-Alvarado et al. 2010). These NSPs demonstrate higher solubility in water. Conversely, feedstuffs abundant in insoluble fibers contain elevated levels of lignin and cellulose, including soybean, rice, oats, various seed hulls, and wheat bran (Slama et al. 2019).

Van Soest et al. (1991) methodology is widely adopted for quantifying insoluble fiber due to its accuracy and reliability in assessing the insoluble fraction of dietary fiber. Cellulose, hemicellulose, and lignin are extracted using a boiling detergent solution with a neutral pH, resulting in the neutral detergent fiber (NDF) fraction. The use of an acid detergent solution solubilizes hemicelluloses, resulting in the residual component referred to as acid detergent fiber (ADF). The crude fiber (CF) refers to organic matter remaining after a series of hot solutions with sulfuric acid and sodium hydroxide acid extractions (Choct 2015).

While CF is utilized to measure the fiber component in poultry diets, it accounts for only a portion of the insoluble NSPs and neglects the soluble fraction. The same applies to ADF and NDF, which are primarily employed in the assessment of forages for ruminants. The determination of NSP necessitates enzymatic hydrolysis of the sample, subsequent precipitation with ethanol, and acid hydrolysis to decompose polysaccharides into monosaccharides. The quantification of released sugars can be conducted utilizing gas chromatography (GLC), liquid chromatography (HPLC), or colorimetric techniques (Englyst and Hudson 1996).

**Fig. 1:**
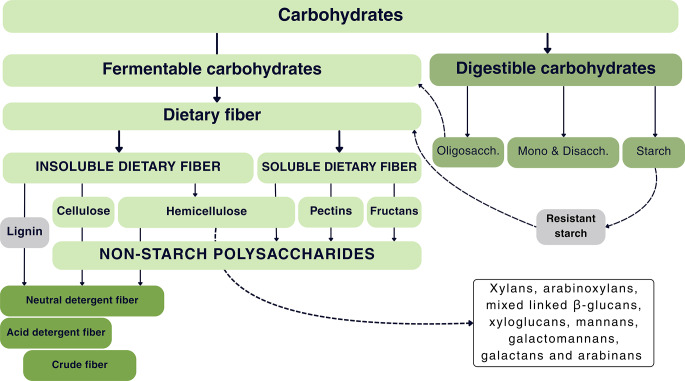
Classification of carbohydrates and the components of dietary fiber

The quantification of DF, although more complex, is more appropriate for describing the fiber content in poultry feed. The insoluble and soluble fiber fractions exert specific effects on the gastrointestinal tract. Thus, the type of fiber consumed can result in different effects concerning feed intake, gastrointestinal motility and development, enzyme synthesis, and the proliferation of intestinal and cecal microbiota (Mateos et al. 2012), which ultimately impact the productive performance of poultry. Figure 1 illustrates the classification of carbohydrates and the constituents of DF.

## Effects on the gastrointestinal tract of poultry

Soluble dietary fiber (SDF) primarily increases the viscosity of the digesta in the small intestine, attributed to the greater water retention capacity (Slama et al. 2019) and the interactions among polysaccharide molecules that create aggregates, which increase the viscosity of the digesta (Choct et al. 1996). The increase in viscosity leads to a reduced digesta passage rate and interferes with the interaction between enzyme and substrate, resulting in decreased digestibility and nutrient absorption (Choct 1997; Svihus and Hetland 2001). The elevated water retention capacity of soluble NSPs results in excreta with increased moisture content (Langhout et al. 1999).

**Fig. 2:**
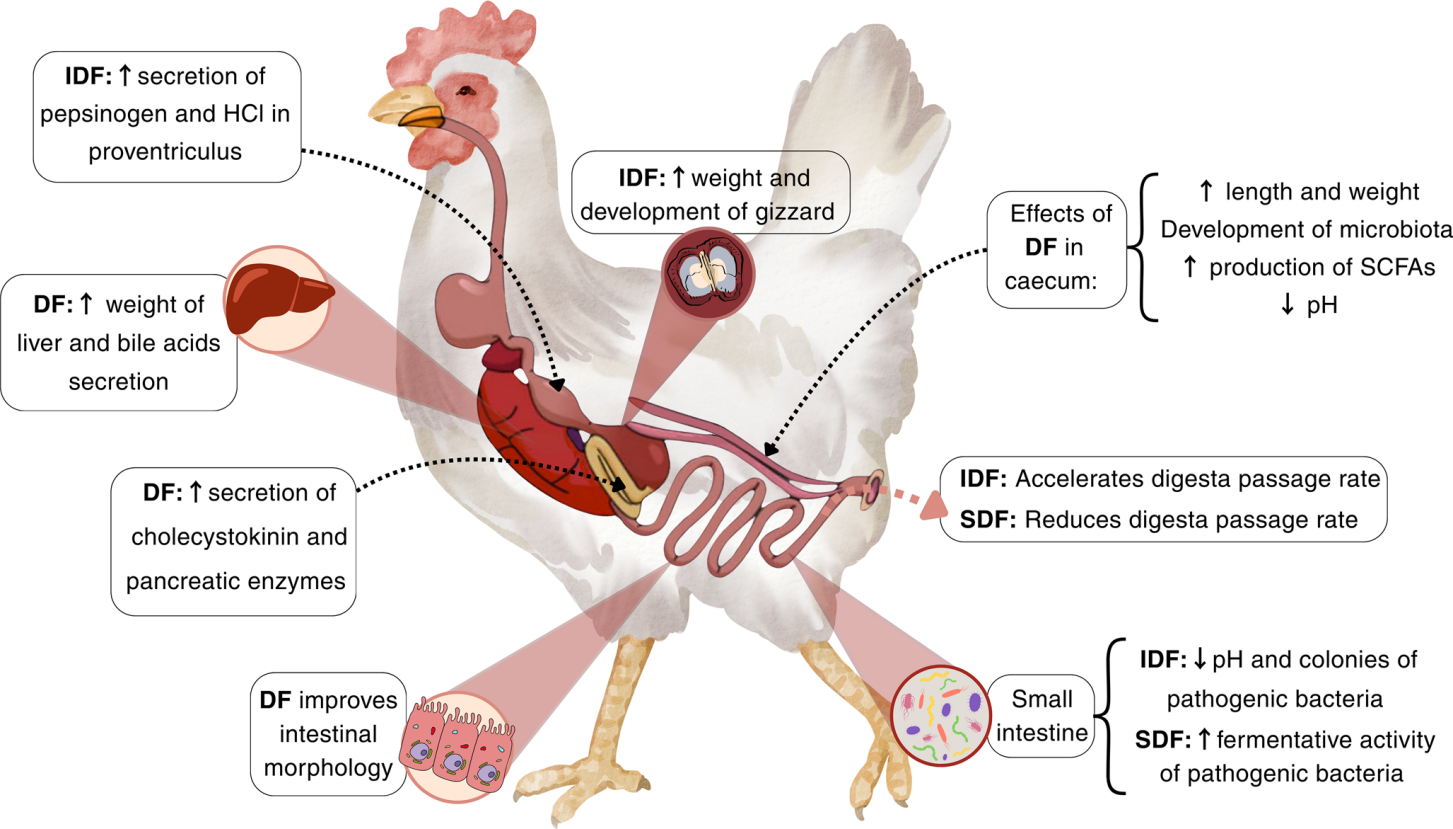
Schematic representation of the physiological effects of dietary fiber (DF) in the gastrointestinal tract of poultry. The figure distinguishes the specific effects of insoluble dietary fiber (IDF) and soluble dietary fiber (SDF). ↑ and ↓ – indicate an increase or reduction in the respective parameters

Insoluble dietary fiber (IDF) exerts a different effect on the gastrointestinal tract relative to the soluble fraction, enhancing the passage rate of digesta and promoting the development and functionality of the digestive organs, particularly the gizzard (Hetland et al. 2003). IDF can reduce the moisture content of the excreta, improving the quality of poultry bedding (Röhe et al. 2020) and decreasing the occurrence of pododermatitis (Naeem et al. 2023). Insoluble fibers not only decrease the moisture content of excreta but also diminish nitrogen excretion, thereby mitigating ammonia emissions into the environment by enhancing amino acid digestibility (Naeem et al. 2023). The main adaptations resulting from dietary fiber consumption are illustrated in Fig. 2, and the effects on the gastrointestinal tract of poultry are summarized in Table 1.

**Table 1:** Effects of dietary fiber in the gastrointestinal tract of poultry

Reference	Source	Species	Level in the diet	Effects
(JøRgensen et al. 1996)	PF, WB or OB	Broiler (12–47 d of age)	PF, WB or OB: 0, 18.7 and 37.5%	PF increased the relative weight of the GIT. The higher level increased the length and weight of the caecum.
(Svihus et al. 2004)	GW or WW	Broiler (11–25 d of age)	GW: 50.0% WW: 37.5%	WW increased the weight of the gizzard, the weight of the pancreas, the secretion of acidic bile, and amylase.
(González-Alvarado et al. 2010)	OH or SBP	Broiler (1–42 d of age)	OH or SBP: 3%	SBP showed a higher weight of the GIT and content in the gizzard. OH presented a higher weight of the gizzard.
(Kalmendal et al. 2011)	HF-SFC	Broiler (15–31 d of age)	HF-SFC: 0, 10, 20 and 30%	Linear reductions of villus height, thickness of muscularis mucosa, and the circular and longitudinal layers of muscularis in the jejunum with HF-SFC inclusion.
(Yokhana et al. 2016)	LC	Laying hen (8 wk of age)	LC: 1%	Increase in the relative weight of the liver, gizzard, small intestine and activities of pepsin, trypsin and pancreatic general proteolytic activity.
(Abdollahi et al. 2019)	LC, OH, WS or WW	Broiler (8–21 d of age)	LC – 1%; OH and WS – 3%; WW – 5 and 10%	Increase in the relative weight of the gizzard and the thickness of the glandular mucosa of the proventriculus.
(Röhe et al. 2020)	LC	Dual purpose hens (52 wk of age)	LC: 10%	Increase weight of the gizzard, small and large intestine, and colorectal surface area.
(Abdollahi et al. 2021)	WB or SBP	laying hens (90 wk of age)	WB or SBP: 3 and 6%	3% de WB increase villus height, WB and SBP reduced cecal pH in 3%.
(Nguyen et al. 2022)	Barley	Laying hen (25 weeks of age)	Soluble NSP: 1.12% vs. 1.34%	The highest level increase the viscosity in the jejunum and the moisture of the excreta.
(Moreno et al. 2023)	DDGS	Broiler quails (1–42 d of age)	DDGS: 0, 5, 10, 15, 20, 25%	Linear increasing of gizzard weight with increasing dietary DDGS levels.

Abbreviations: PF – Pea fibre; WB – Wheat bran; OB – Oat bran; GTI – Gastrointestinal tract; GW – Ground wheat; WW – Whole wheat; OH – Oat hull; SBP – Sugar beet pulp; HF-SFC – High-fibre sunflower cake; LC – Lignocellulose; WS – Wood shavings; NSP – Non-starch polysaccharide; DDGS – Distillers’ dried grains with solubles

Birds cannot fully digest NSPs due to the lack of endogenous hydrolyzing enzymes (Zhang et al. 2022). The GIT microbiota facilitates the digestion of NSPs through fermentation. Nonetheless, the degradation is less effective than in other monogastric animals, resulting in the fermentation and utilization of NSPs at a low proportion due to the relatively short caecum (JøRgensen et al. 1996). The products of bacterial fermentation are primarily short-chain fatty acids (SCFAs), consisting of two to six carbon atoms, including acetate, propionate, and butyrate. Fermentation products lower intestinal pH, promoting the proliferation of *Lactobacillus spp.* while inhibiting pathogenic bacteria, and confer beneficial effects on intestinal cells (Williams et al. 2017). Butyrate serves as an energy substrate for intestinal cells and is crucial for sustaining intestinal hypoxia via oxygen consumption through β-oxidation, thereby preventing dysbiosis of the intestinal microbiota. Propionate modulates gluconeogenesis, while acetate serves as an energy and carbon source for the proliferation of other bacteria and is crucial in cholesterol metabolism and lipogenesis (Valdes et al. 2018).

The SCFAs generated can provide a minor portion of dietary energy, as evidenced by the research conducted by JøRgensen et al. (1996). This study indicates that broiler chickens consuming diets with varying levels of pea fiber, wheat bran, or oat bran derived approximately 3 to 4% of their metabolizabe energy from the fermentation of NSPs. However, the degree of fermentation depends on the type of DF, with the insoluble fraction undergoing low fermentation. The IDF fraction remains largely undigested until it arrives at the caecum, where fermentation occurs (JøRgensen et al. 1996). Conversely, SDF is fermented more rapidly and can occur in both the small intestine and the caecum (Williams et al. 2017). Since soluble NSPs undergo fermentation before reaching the caecum, an increase in the intake of these polysaccharides causes the proliferation of fermentative bacteria in the small intestine, negatively affecting nutrient digestibility and the reduction of ME of the diets (Choct et al. 1996).

The increased intake of DF stimulates the growth of digestive organs, as evidenced by JøRgensen et al. (1996), wherein increasing inclusions (0, 18.7, and 37.5%) of pea fiber, wheatbran, or oat bran in broiler chickens led to an increase in both the length and weight of the caecum, attributable to higher activity and development of caecal microbiota. The length of the small intestine was more pronounced with pea fiber, which has a higher proportion of soluble NSPs, producing greater distension due to its higher water retention capacity and reduced digesta passage rate.

González-Alvarado et al. (2010) achieved similar results by including 3% oat hull (high in insoluble fibers) and sugar beet pulp (high in soluble fibers) for broiler chickens. Both fiber sources augmented the weight of the GIT organs relative to the control. However, the increase in organ weight and contents was more pronounced with sugar beet pulp, suggesting greater distension linked to a reduced passage rate attributable to the high pectin content in this feedstuff. Another alteration was observed in the gizzard, which exhibited increased size and weight when fed oat hull instead of sugar beet pulp. This change is due to the particles of oat hull having a high degree of lignification, making them more efficient in promoting the development of gizzard than soluble fibers.

Insoluble fiber-rich feedstuffs can variably influence gizzard development based on the source utilized. The incorporation of wood shavings led to an increased relative weight of the empty gizzard in broilers in comparison to diets containing cellulose or whole wheat (Amerah et al. 2009). Abdollahi et al. (2019) observed similar findings, indicating an increased relative weight of the empty gizzard with the incorporation of 3% wood shavings (25.1 g/kg of body weight) and oat hull (24.2 g/kg) in comparison to whole wheat (18.7 g/kg), while lignocellulose exhibited the lowest value among the insoluble fiber sources (14.9 g/kg). Consequently, particles that exhibit greater resistance to grinding appear to be retained in the gizzard for a longer period, promoting its development. Additionally, diets with a greater average geometric diameter promote gizzard development. However, the adaptation resulting from the inclusion of insoluble fibers is more significant (Abdollahi et al. 2019), and the elevation of insoluble fiber levels leads to a linear increase of gizzard weight (Moreno et al. 2023).

The proventriculus is another organ that experiences adaptations with the incorporation of fiber. The increase in weight is associated with the enhanced feed retention capacity of the gizzard and the modification in the transit rate of digesta through the GIT (Li et al. 2017; Röhe et al. 2020). Abdollahi et al. (2019) observed no variation in weight with the utilization of diverse sources of insoluble fiber. However, the addition of fibers caused an increase in the thickness of the glandular mucosa of the proventriculus. In the proventriculus, hydrochloric acid and pepsinogen are secreted, and due to the small capacity of the organ, the digesta remains for a short period, so the digestive action of these secretions mainly proceeds in the gizzard (Svihus 2011). In response to the contractions of the gizzard muscles, the digesta refluxes back into the proventriculus, stimulating additional secretions of hydrochloric acid and pepsinogen (Duke 1992).

The consumption of fiber, particularly IDF, can inhibit the adherence of pathogenic bacteria to the intestinal epithelium due to the abrasive effect caused by the particles on the mucosa (Jha and Mishra 2021). This may result in higher mucus production by goblet cells, thereby enhancing the mucus layer between the intestinal lumen and the microvillus, which protects enterocytes from mechanical injury induced by the fiber particles (Langhout et al. 1999). However, despite the thicker layer, fiber can cause damage to the mucosa and produce an inflammatory response in the intestinal epithelium (Teirlynck et al. 2009).

SDF tends to increase the length of the small intestine due to its high solubility and reduced passage time (JøRgensen et al. 1996; González-Alvarado et al. 2010; Abdollahi et al. 2021). On the other hand, the insoluble fraction may show a reduction in intestinal length due to the diet dilution effect, so a smaller absorption area is needed, with the reduction in length being compensated by a larger intestinal diameter, which increases the absorption area (Amerah et al. 2009). Kalmendal et al. (2011) observed a linear decrease in the thickness of the muscular mucosa of the jejunum resulting from intestinal distension caused by the progressive inclusion of IDF in the diet.

Intestinal morphology also undergoes changes with the inclusion of fiber in the diet, as demonstrated by Tüzün et al. (2021) for broiler chickens in the initial and growth phases, where the inclusion of sunflower meal (with a high proportion of insoluble fiber) at the highest fiber level (between 5.1 and 5.9%) showed worse reults for villus height and width, and a lower ratio villus height:crypt depth compared to low (2.9 and 3.1%) and medium fiber (4.0 and 4.5%) diets. The intemediate fiber level demonstrated improvement in intestinal morphology, suggesting that moderate amounts can improve the absorptive capacity of the small intestine. Conversely, other research did not identify alterations with the incorporation of diverse sources or levels of fiber for broilers (Langhout et al. 1999; Abdollahi et al. 2019). Abdollahi et al. (2021) conducted a study on 70-week-old laying hens, revealing that the inclusion of 3% wheat bran enhanced the height of the jejunal villi, the villus height:crypt depth rato, and the surface area of the villi.

The intestinal microbiota can be modified by the inclusion of fiber in poultry diets, as indicated by Teirlynck et al. (2009), where diets containing rye and/or barley resulted in significant alterations of the microbiota compared to zinc bacitracin supplementation. Kalmendal et al. (2011) observed a significant reduction in *Clostridium spp.* colonies through the addition of sunflower cake with a high proportion of insoluble fiber. The inclusion of 3% citric pectin in chickens led to an elevatin in the overall count of aerobic bacteria, as well as bacteria from the genera *Enterococcus spp.*, *Bacteroidaceae*, *Clostridia spp.*, and *E. coli*, in comparison to the control group (Langhout et al. 1999). Since the ingredient used has high solubility, there is an increase in the viscosity of the digesta, which implies a reduction in digestibility by the bird’s endogenous enzymes. Thus, a greater amount of undigested nutrients is available in the distal part of the small intestine for bacterial growth and fermentation.

In addition to the small intestine, the caecal microbiota is affected by the fiber level, such that low-fiber diets (2.5% CF) can decrease the diversity of the caecal microbiota and reduce the number of beneficial caecal colonies compared to high-fiber diets (6.1%), impacting fiber digestibility (Li et al. 2018). The addition of dietary sources of insoluble or soluble fiber can cause a reduction in cecal pH, indicating increased fermentative activity (Abdollahi et al. 2021). An interesting result was found by Röhe et al. (2020) with the inclusion of 10% lignocellulose in layer diets. The control treatment (0% lignocellulose) exhibited a greater concentration of bacterial fermentation products than the 10% inclusion. However, these results do not indicate that the fermentation of lignocellulose was reduced, as the birds that consumed lignocellulose showed a greater surface area of the intestinal mucosa due to the increase in villi and crypts in the colorectal region. These results indicate that the inclusion of fiber can improve the absorptive capacity of bacterial fermentation metabolites through the increase in surface area, which occurs due to the increase in length of the caecum.

## Dietary fiber can improve nutrient utilization

As previously discussed, fiber causes different adaptations and effects in the gastrointestinal tract of poultry, so these changes reflect on nutrient digestibility, depending on the composition of the NSPs in the diet (Table 2).

**Table 2:** Effects of dietary fiber on the utilization of energy and nutrients in the diet of poultry

Reference	Source	Species	Level in the diet	Effects
(Choct et al. 1996)	Soluble NSPs extracted from wheat	Broiler (21–29 d of age)	Soluble NSP: 40 g/kg	NSP reduced the digestibility of starch, CP, EE, and AME. Increased the moisture of the excreta and the viscosity of the digesta.
(JøRgensen et al. 1996)	PF, WB or OB	Broiler (12–47 d of age)	PF, WB or OB: 0, 18.7 and 37.5%	Reduction in the digestibility of DM, NSP, and energy with the addition of fiber sources.
(Svihus et al. 2004)	GW or WW	Broiler (11–25 d of age)	GW: 50.0% WW: 37.5%	WW increased the digestibility of energy and starch.
(González-Alvarado et al. 2010)	OH or SBP	Broiler (1–42 d of age)	OH or SBP: 3%	Increase in the digestibility of DM, ash, N, EE, and AMEn.
(Kalmendal et al. 2011)	HF-SFC	Broiler (15–31 d of age)	HF-SFC: 0, 10, 20, 30%	Linear increases in ileal digestibility of EE and CP and linear decreases in DM, ash and energy with HF-SFC inclusion.
(Abdollahi et al. 2019)	LC, OH, WS or WW	Broiler (8–21 d of age)	LC – 1%; OH and WS – 3%; WW – 5 and 10%	Effect on the digestibility of energy, DM, starch, and Ca.
(Nguyen et al. 2022)	Barley	Laying hen (25 weeks of age)	Soluble NSP: 1.12% vs. 1.34%	The higher level reduced the energy digestibility and increased the NSP digestibility.
(Leite et al. 2024)	LC	Broiler (21 d of age)	LC: 1, 2 and 3%	The level of 1% increased the ileal digestibility of EE, CP and amino acids.
(Suyama et al. 2025)	Kikuyu grass or Annual ryegrass	Laying hen (72 and 95 weeks of age)	Kikuyu grass or Annual ryegrass: 3, 6 and 9%	A linear decrease in AME, digestibility of DM, energy, and NDF. The digesta passage time reduced linearly with the increase in the level of grass.

Abbreviations: NSP – Non-starch polysaccharide; CP – Crude protein; EE – Ether extract; PF – Pea fibre; WB – Wheat bran; OB – Oat bran; DM – Dry matter; GW – Ground wheat; WW – Whole wheat; OH – Oat hull; SBP – Sugar beet pulp; HF-SFC – High-fibre sunflower cake; LC – Lignocellulose; WS – Wood shavings; NDF – Neutral detergent fiber

Angkanaporn et al. (1994) conducted a study with male chickens and evaluated the digestibility and endogenous loss of amino acids using pentosans (soluble NSPs) extracted from wheat at levels of 1.5% and 3.5% and insoluble fiber sorces with the addition of 9.2% in the diet. Even the ddition of the lower level of pentosans was able to significantly reduce digestibility and increase endogenous amino acid loss compared to the control and treatments with insoluble fiber. The effect caused by insoluble fibers was lower, despite being added at higher levels. By increasing the viscosity of the digesta, pentosans can interfere with the passage of feed, creating greater resistance to intestinal peristalsis and interfering with the action of peptide hormones, which can increase the secretion of endogenous proteins.

Another deleterious effect of soluble NSPs is the influence they have on the digestibility of EE (Langhout et al. 1999). The inclusion of citrus pulp pectins caused a reduction in EE digestibility and, consequently, in ME in chicken diets. The reduction in digestibility occurred indirectly through the increase of fermentative bacteria, as some species of bacteria are capable of deconjugating bile acids, interfering with the digestibility of fatty acids. Bile acids are fundamental in the emulsification of long-chain or saturated fatty acids, facilitating the action of lipases and, consequently, the formation of micelles that can be absorbed in the small intestine (Smits and Annison 1996).

Studies using insoluble fibers show an increase in the digestibility of some nutrients, such as calcium (Abdollahi et al. 2019) and starch (Hetland et al. 2003; Svihus et al. 2004; Amerah et al. 2009; Abdollahi et al. 2019), but divergent results for the digestibility of dry matter (DM), protein, EE, and ME (González-Alvarado et al. 2010; Kalmendal et al. 2011; Li et al. 2017; Abdollahi et al. 2019; Leite et al. 2024; Suyama et al. 2025). Such results are mainly due to the development of the gizzard, which performs various functions in the gastrointestinal tract, such as grinding and reducing particles before they are released into the duodenum and acting in the regulation of motility and the control of digesta flow and gastroduodenal reflux. Furthermore, the gizzard is related to the secretion of digestive enzymes, such as bile acids, endogenous enzymes, and hydrochloric acid (Mateos et al. 2012).

In addition to causing the development of gizzard, insoluble fibers can increase the relative weight of the liver and pancreas (Iqbal et al. 2018). The liver produces bile acids and is crucial for the metabolism, synthesis, and storage of dietary lipids, carbohydrates, and proteins. The increase in the size of the liver and pancreas is related to the increase in the secretion of bile acids and digestive enzymes, as observed by Svihus et al. (2004), who found an increase in the digestibility of starch and ME in the diet of chickens using whole wheat instead of ground wheat, and there was an increase in the activity of amylase and bile acids in the jejunum, which explains the improvement in the nutritional value of the feed with the use of whole wheat. Hetland et al. (2003) also observed an increase in starch digestibility in broilers and layers using insoluble fibers, with an increase in the concentration of amylase and bile acids in the jejunum.

Cholecystokinin, a gastrointestinal hormone synthesized and excreted by endocrine cells primarily present in the duodenum, has important digestive functions related to the improvement of digestion through the contraction of the gallbladder, secretion of pancreatic enzymes, intestinal motility, and regulation of feed intake (Wan et al. 2023). Cholecystokinin acts by stimulating the vagus nerve for the secretion of digestive enzymes by the exocrine pancreas (Li and Owyang 1993) and acts on gastroduodenal reflux (Duke 1992). Insoluble fibers seem to modulate the secretion of cholecystokinin, and the increase in its activity may be related to the structural and functional development of the gizzard. The studies conducted by Hetland et al. (2003) and Svihus et al. (2004) support this finding, as IDF diets promoted the development of the gizzard associated with an increase in the secretion of pancreatic enzymes and bile acids. Furthermore, the authors observed an increase in bile acids in the gizzard, indicating the occurrence of gastro-duodenal reflux, as bile is released in the posterior portion of the duodenal loop, caused by the increased secretion of cholecystokinin. The increase in bile acids in the gizzard can improve the emulsification of dietary fats and enhance the solubilization of other nutrients, such as starch, and consequently, their digestibility (Hetland et al. 2003).

The utilization of dietary calcium can be improved through the addition of insoluble fiber sources, as demonstrated by Abdollahi et al. (2019). The authors utilized various fiber sources, including lignocellulose, oat hulls, wood shavings, and whole wheat, with a 3% substitution relative to a control diet in mash form for broiler chickens. The inclusion of fibers significantly increased the digestibility of calcium compared to the control diet. However, there was no statistical difference between the evaluated sources, with digestibility ranging from 44.4% to 47.0%, while the control showed a digestibility of 29.2%. The authors also found an increase in the thickness of the glandular mucosa of the proventriculus and a relative increase in the gizzard with the inclusion of fiber sources. These two adaptations of the GIT can explain the improved utilization of calcium by the birds, as the proventriculus and the gizzard work together as a single structure in the gastric digestion process.

The digestive efficiency of hydrochloric acid and pepsinogen secreted by the proventriculus is closely related to the functionality of the gizzard in terms of contraction intensity, reduction of digesta particles, and, most importantly, retention time, which is increased by the inclusion of highly lignified fibers (Svihus 2011). And, as demonstrated by Guinotte et al. (1995), gastric secretions play an important role in the solubilization of calcium, which is facilitated by the reduction of pH and the consequent activation of pepsinogen, thus favoring the intestinal absorption of this mineral.

The addition of IDF causes a more prominent reduction in pH in the upper portion of the gastrointestinal tract (Gabriel et al. 2003 zün et al. 2021). The pH of the gizzard can vary from 1.9 to 4.5 (Svihus 2011). In a study conducted by Gabriel et al. (2003) with whole wheat for chickens, the pH of the gizzard was reduced to 3.31 compared to a diet without the addition of wheat, which showed a value of 3.99, resulting in a reduction of pepsinogen activity in the proventriculus tissue of birds fed with whole wheat compared to the control (76 ± 13 vs. 96 ± 12 units/g of body weight). These results indicate that there was a greater stimulus for gastric secretion in the birds fed with wheat.

The reduction of the pH of the gizzard contents, associated with increased pepsin activity, can enhance the denaturation and hydrolysis of dietary proteins (Gabriel et al. 2003). Thus, the digestibility of protein can be improved using insoluble fibers in the diet (González-Alvarado et al. 2010; Kalmendal et al. 2011; Li et al. 2017; Suyama et al. 2025) and increase the ileal digestibility of amino acids (Angkanaporn et al. 1994; Naeem et al. 2023). Another benefit of reducing pH is the bactericidal effect, which can cause a reduction in colonies of pathogenic bacteria (Svihus 2011), in addition to the indirect effect through the improved utilization of nutrients due to increased gastric secretion and gizzard activity, so that intestinal absorption is enhanced and a smaller amount of undigested particles will be available for the growth of pathogenic bacteria (Gabriel et al. 2003).

In general, diets with moderate inclusion of fibers can improve nutrient utilization. Both soluble and insoluble fibers have positive effects on nutrient digestibility. González-Alvarado et al. (2010) observed that the inclusion of 3% sugar beet pulp or oat hulls for broilers increased the utilization of DM, organic matter (OM), nitrogen retention, EE, ME, and soluble ash. Higher values were obtained for DM, OM, and N retention with oat hulls. Diets with low fiber (2.5%) are associated with an underutilization of energy and crude protein when compared to diets with intermediate (4.3%) or high (6.1%) CF levels, hich can impact the productive performance of the birds (Li et al. 2017). However, high inclusions of fibrous feedstuffs (18.7 or 37.5%) rich in soluble or insoluble fibers can redce the digestibility of dietary nutrients, as demonstrated by JøRgensen et al. (1996), because poultry have a limited capacity to digest DF due to a deficiency of endogenous digestive enzymes and reduced fermentative capacity.

## Reduction of anti-nutritional effects of soluble NSPs using carbohydrases

The main constituents that represent the majority of diet expenses are ingredients sources of energy and protein, commonly using grains such as corn, wheat, and sorghum, as well as plant-based meals. Nonetheless, the grains constituting these ingredients are utilized in other industrial processes, including biofuel production, thus, their availability and rising value may impede their application in poultry feed. One alternative is the utilization of alternative feedstuffs. However, these ingredients generally contain elevated levels of NSPs that impact digestion. In order to overcome this issue, carbohydrases can improve the efficiency of the digestive process and the energy utilization of feedstuffs rich in NSPs (Choct 2006).

Carbohydrases are synthesized by bacteria and fungi. These microorganisms can produce enzymes like cellulases, β-glucanases, and xylanases, which hydrolyze the β bonds in the chains of NSPs (Raza et al. 2019), facilitating greater breakdown of these polysaccharides, since birds are not capable of synthesizing endogenous enzymes to hydrolyze the β bonds.

The main NSPs that make up dietary fiber in foods are β-glucans, cellulose, and hemicellulose. β-glucans consist of glucose polymers linked by β1–3 and β1–4 bonds (Bach Knudsen 2001). Cellulose consists of a linear chain of glucose linked by β1–4 glycosidic bonds (Choct 2006). Hemicellulose (arabinoxylans) consists of a linear chain of xylose units linked by β1–4 bonds, with side chains of various sugars, predominantly arabinose (Raza et al. 2019). Xylanases and β-glucanases are the primary carbohydrases employed for the degradation of hemicellulose and β-glucans, respectively. Cellulose necessitates a combination of three distinct enzymes - endo-glucanase, exo-glucanase, and β-glucosidase - that target different segments of the polymer chain for its complete degradation (Choct 2006). However, carbohydrases are not capable of completely breaking down NSPs into simple sugars, as birds have a short digesta passage time and relatively short caecum, limiting the action capacity of carbohydrases in the digestion of NSPs (de Vries et al. 2012).

Starch may not be completely digested at the end of the ileum, and the undigested portion is referred to as resistant starch. Upon reaching the caecum, resistant starch is fermented, producing acetate and lactate. However, large quantities of starch available for fermentation can be harmful to birds. Thus, the use of the enzyme amylase can increase its degradation in the small intestine (Bedford and Apajalahti 2022).

Several studies demonstrate the benefits of using carbohydrases, and there are four main mechanisms of action in the digestive tract of birds: (1) the degradation of soluble NSPs, causing a reduction in the viscosity of the digesta in the duodenum, jejunum, and ileum (Choct et al. 1996; Nguyen et al. 2022); (2) reduction of bacterial fermentation in the jejunum and ileum, due to a lower amount of polysaccharides available for bacterial development (Choct et al. 1996; Bedford and Apajalahti 2022); (3) they can degrade complex polysaccharides into simpler sugars, which can be utilized by the bacteria present in the caecum, resulting in greater production of SCFAs (Singh et al. 2019); (4) they can degrade the plant cell wall, allowing the action of endogenous and/or exogenous digestive enzymes on the nutrients present inside the plant cell, such as proteins, starch, and phosphorus (Singh et al. 2019). Moreover, they can decrease the moisture content of excreta in diets high in soluble NSP, owing to the greater breakdown of these polysaccharides (Nguyen et al. 2022).

A study conducted by Meng et al. (2005) using carbohydrases individually or in combination for chickens indicated better results in the digestibility of starch, protein, and energy through the combination of carbohydrases, which can be explained by the greater degradation of NSPs and the reduction of viscosity. These results demonstrate that the enzyme combination is more effective in enhancing dietary nutrient utilization, as each enzyme targets a specific substrate, resulting in a synergistic effect among the utilized enzymes. The combination of carbohydrases with proteases and/or phytase can improve the digestibility of protein and phosphorus, respectively, as the deleterious effects of NSPs can be mitigated, associated with a greater release of nutrients from the interior of the plant cell (Amerah et al. 2017).

## Dietary fiber and productive performance

In formulating poultry diets, minimal inclusion of feedstuffs rich in DF is prioritized to prevent the occurrence of their antinutritional effects. Nonetheless, as previously mentioned, the GIT can adapt to increased intake of NSPs, improving nutrient utilization, thereby lowering feed formulation costs and minimizing nutrient excretion. Most research emphasizes the incorporation of various feedstuffs rich in soluble and insoluble NSPs, rather than the concentration of DF in diets, to evaluate their impact on digestibility, productive performance, and alterations in the GIT. This focus is justifiable considering the distinct effects of the soluble and insoluble fractions, as well as the influence of particle size of the ingredient.

In laying hens of two different strains (Hy-Line Brown and Lohmann LSL), increasing NDF concentration from 14.5% to 18.5% during the growth phase (7 to 12 weeks of age) did not affect carcass composition, bone quality, feed intake, or weight gain in isoenergetic and isoproteic diets. However, the level of NDF increased the relative weight of the liver and intestines (Freitas et al. 2014). These results indicate that birds can adapt their GIT to digest diets with higher levels of NDF and absorb the nutrients and energy necessary for their growth, allowing the use of alternative feedstuffs - which usually have higher levels of NSPs - and a greater range for fiber content in diet formulation. However, it should be considered that feedstuffs rich in fiber have lower ME values, requiring the inclusion of feedstuffs with higher energy density to meet nutritional requirements.

Han et al. (2023) assessed diets with NDF concentrations between 9.01% and 13.42% fo 75-week-ol laying hens. The rise in NDF levels augmented feed consumption, yet other productivity metrics remained unaffected. The diet with elevated NDF reduced hepatic fat accumulation and the expression of *FASN*, *ACC*, *SCD*, *PPAR-γ*, and malic enzyme, which are associated with fatty acid synthesis. Dietary fiber may suppress de novo fatty acid synthesis, reduce hepatic lipogenesis and triglyceride production, and increase lipoprotein lipase activity in adipose tissue, thereby facilitating a reduction in fat accumulation in the liver and plasma (Akiba and Matsumoto 1982). Minimizing fat accumulation in the liver can decrease the incidence of fatty liver hemorrhagic syndrome (FLHS), a significant metabolic disorder in poultry, predominantly impacting caged laying hens that display excessive hepatic and abdominal fat deposition. The etiology of FLHS remains ambiguous, however, the accumulation of hepatic fat results from lipogenesis rather than dietary fat (Zaefarian et al. 2019).

A study conducted by (Abdollahi et al. 2021) on 70-week-old laying hens supplemented with 3% or 6% wheat bran or sugar beet pulp observed that both levels of sugar beet pulp inclusion and 6% wheat bran caused a reduction in feed intake, while the 3% wheat bran level did not differ from the control (0%). Only the 6% inclusion of wheat bran and both levels of sugar beet pulp resulted in a decrease in egg production, while no impact was noted on egg weight, feed conversion, or egg quality with the incorporation of these feed ingredients. These findings illustrate the detrimental impact of soluble NSPs, which diminish feed intake and egg production by increasing digesta viscosity and subsequently reducing diet digestibility.

In a comparable study involving broiler chickens, conducted by González-Alvarado et al. (2010), a 3% substitution of sugar beet pulp or oat hulls revealed that oat hulls enhanced protein and energy digestibility, daily weight gain, final weight, and feed conversion over the entire evaluation period of the experiment (1 to 42 days of age) in comparison to the control group. Sugar beet pulp demonstrated a decline in growth performance after 25 days of age, suggesting that feedstuffs high in insoluble NSPs are more effective in increasing dietary fiber concentration in poultry diets. Furthermore, the increased consumption of feed as the chickens aged elevated the amount of soluble NSPs in the GIT, contributing to the manifestation of their antinutritional effects.

Naeem et al. (2023) performed a study on broiler chickens with a 5% substitution of oat hulls with average geometric diameters of 400 and 850 μm, compared to a reference diet. The substitution of oat hulls showed no significant difference in final body weight, body weight gain, and feed consumption compared to the control group. Only feed conversion worsened with the smaller particle size and the larger particle size did not differ from the control. The gizzard exhibited increased weight, relative weight, and an elevated gizzard:proventriculus ratio in the treatment involving coarser oat hulls. Despite the reduction of energy and protein in the diet, the birds exhibited comparable productive performance when oat hulls of larger particle size were utilized. The findings suggest that insoluble fiber can enhance the utilization of energy and nutrients from diets. The enhanced digestibility of energy and nutrients allows for the formulation of feed with reduced energy and protein densities, thereby decreasing diet costs and nitrogen excretion into the environment through improved nutrient utilization.

Birds with shorter production cycles appear to benefit more from the addition of dietary fiber, due to the greater development of the GIT - improving digestive capacity and nutrient absorption - which has not yet reached full maturity by the time of slaughter. However, one of the major challenges of including DF in poultry diets is determining concentrations that do not compromise the digestive process and that may negatively affect the productive performance of the birds, since, as discussed, SDF has deleterious effects, and even low inclusions of feedstuffs rich in soluble NSPs can compromise the productive performance.

## Conclusion and perspectives

Additional research is necessary for determining adequate nutritional levels of dietary fiber, as there are currently no recommended concentrations for poultry diets, including CF, probably due to the negative relationship between fiber and digestibility as well as productive performance in poultry. However, moderate inclusions of fiber, especially from insoluble sources, can promote adaptations in the gastrointestinal tract and improve feed utilization by increasing digestive secretions, gizzard capacity and functionality, and intensifying the fermentative activity of bacteria. Some non-traditional ingredients in poultry feed have higher levels of NSPs and can be used in diets due to the beneficial effect of dietary fiber on the gastrointestinal tract and their lower cost. The incorporation of fiber enhances nutrient and energy absorption from diets, potentially lowering feed expenses and promoting sustainability through diminished environmental nutrient excretion. The deleterious effects caused by soluble NSPs, which include increased viscosity and bacterial fermentation in the small intestine, can be mitigated using exogenous carbohydrases. These carbohydrases help break down the NSPs, reducing viscosity and promoting better nutrient absorption in the small intestine.

## Data Availability

All available data is included in the manuscript.
